# Evaluation of recombinant human vascular endothelial growth factor VEGF121-loaded poly-l-lactide microparticles as a controlled release delivery system

**DOI:** 10.3906/biy-1908-32

**Published:** 2020-02-17

**Authors:** Sunil ABRAHAM, Srinivasa Prasad RANGASWAMY, Amutha CHINNAIAH

**Affiliations:** 1 Department of Animal Behavior and Physiology, School of Biological Sciences, Madurai Kamaraj University, Madurai India; 2 Innov4Sight Health and Biomedical Systems Biologics Laboratory, Karnataka India

**Keywords:** VEGF, purification, poly-l-lactide, human umbilical vein endothelial cells, proliferation, cell migration, CAM assay, sustained release

## Abstract

Vascular endothelial growth factor A (VEGF-A) is an important growth factor that plays a major role in angiogenesis. With different isoforms distributed in various tissues, the shortest isoform of VEGF-A is VEGF121, one of the physiologically functional variants next to VEGF165. It is well known that VEGF has a shorter half-life, and the stability of the protein must be considered in therapeutic aspects. Poly-l-lactide (PLA) microparticles can release the encapsulated protein in a sustained release mode. In this study, the VEGF121 gene was cloned and expressed in a prokaryotic expression system (*Escherichia coli*). The recombinant VEGF121 was encapsulated with PLA microparticles and studied *in vitro *and *ex ovo *for the sustained release mechanism. The PLA-VEGF microparticles and the recombinant VEGF121 were explored for their bioactivity in human umbilical vein endothelial cells (HUVEC). VEGF released *in vitro *from PLA microparticles on days 1, 20, and 30 showed remarkable biological activity compared to PBS-loaded PLA microparticles such as the ability of the cells to proliferate, migrate, and form tubes similar to recombinant VEGF121. Besides, PLA-VEGF microparticles and the recombinant VEGF121 were also tested for their proangiogenic action in embryonated eggs by chicken chorioallantoic membrane assay (CAM), and the effect was observed in both forms. This study suggests that PLA-loaded VEGF microparticles in a sustainable release format can be effectively used in proangiogenic therapy and reduce the adverse effects caused due to multiple dosages.

## 1. Introduction

Angiogenesis is the formation of blood vessels from existing vasculature in the body. This physiological process begins from birth and continues throughout life in both healthy and diseased individuals (Adair and Montani, 2010). Vascular endothelial growth factor (VEGF) or vascular permeability factor (VPF) (Senger et al., 1983; Ferrara and Henzel, 1989) is an important protein helpful in the formation of blood vessels, and it also plays an important regulatory mechanism in physiological and pathological process of angiogenesis (Folkman and Klagsbrun, 1987; Nowak et al., 2010). VEGF is a heparin-binding growth factor (Leung et al., 1989) and an effective mitogen for endothelial cells (Walsh et al., 2002). VEGF-A, VEGF-B, VEGF-C, VEGF-D, PGF (Placental Growth Factor), and VEGF-E comprise proteins in the VEGF family (Shibuya, 2011), which are present in human genomes (Shibuya, 2011). These entire proteins share conserved 8 cysteine residues (Shibuya, 2011; Taktak-Ben Amar et al., 2017), among which 2 cysteine residues contribute to homodimer structure and the other six generate three-loop structures (Muller et al., 1997; Shibuya, 2011). VEGF A binds to the receptors VEGFR-1 and VEGFR-2, activates the angiogenesis pathways (Taktak-Ben Amar et al., 2017), and helps in cell migration and vascular permeability (Shibuya and Claesson-Welsh, 2006). VEGF A has different splice variants such as VEGF121, VEGF165, VEGF189, and VEGF206, depending upon the variations in amino acids (Ferrara et al., 2003; Ferrara and Kerbel, 2005; Taktak-Ben Amar et al., 2017). In 1998, Huang et al. found that endometrial stromal cells (the region where angiogenesis takes place during the mid to late proliferative phase) have an abundant expression of VEGF121 **(**Huang et al., 1998). The molecular weight of VEGF121 (the isoform used in this study) in homodimer form is 28 kDa and 16kDa in monomer form (Kim et al., 2007). VEGF121 signals through the VEGF receptor 2 (VEGFR2) and is capable of increasing vessel diameter (Nakatsu et al., 2003). During appropriate administration, VEGF can promote angiogenesis and signal vascular endothelial cells which help in cell proliferation and migration (Geng et al., 2011). Several clinical trials on proangiogenic growth factor delivery have been successful (Makinen et al., 2002; Rissanen et al., 2003; Adair and Montani, 2010; Rui et al., 2012) while double-blinded clinical trials on intravenous infusions of recombinant human VEGF failed to show efficacy in a large group of patients. This could be due to the instability or short half-life of the protein. (Simons et al., 2002; Henry et al., 2003; Rui et al., 2012).** **Although the therapeutic effects of this protein can be achieved at high doses, it may lead to the development of vascular tumors (Lee et al., 2000; Geng et al., 2011). Thus, it is mandatory to provide a controlled delivery mechanism to release the bioactive molecules directly to the desired site. VEGF-loaded microparticles in a sustained release form increased retinal vascular remodeling (Mezu-Ndubuisi et al., 2019). PLA microparticle formulations available in the market for clinical use include Lupron Depot and Zoladex (endometriosis and prostate cancer applications), Nutropin Depot (pediatric growth hormone deficiency), and Bydureon (Type 2 diabetes) (Lee et al., 2016). Thus, rVEGF will be efficient in combination with lactate (PLA), which could be effectively used for tissue remodeling, vasculogenesis, and angiogenesis.

In this study, we propose to explore the use of poly-l-lactide (PLA) as a delivery system for VEGF121 and characterize the chemical properties of the PLA microparticle. This system was investigated for surface appearance and shape by scanning electron microscopy (SEM) and drug release kinetics. Analysis of the bioactivity of VEGF released from the PLA particle was performed by testing in human umbilical vein endothelial cells (HUVEC) for proliferation and wound healing assay. Additionally, angiogenesis assay was performed in the chicken chorioallantoic membrane (CAM). The ultimate goal of this study is to identify the bioavailability and stability of VEGF microspheres as potential candidates for a localized delivery system for improving therapeutic efficacy. 

## 2. Materials and methods

### 2.1. VEGF121 construct preparation

The total RNA was extracted from Immortalized Human Endometrial Stromal Cells (HESC) (ABM, Canada) using an RNA easy kit (RNeasyMinikit, Qiagen Inc., Valencia, USA) as per the manufacturer’s procedure. RT–PCR was performed for the first strand synthesis using a Thermo Scientific Revert Aid First Strand cDNA Synthesis Kit using random Hexa primers. The VEGF121 fragment was amplified from the cDNA clone by PCR using *Pfu* DNA polymerase (MBI, Fermentas, MD, USA) and VEGF121 gene-specific primers VGFF (NdeI): 5’CATATGGCACCCATGGCAGAAGGAGG3’ and VGFR (BamHI): 5’GGATCCCCGCCTCGGCTTGTCACATC3’. The amplification was carried out in a T100Tm Thermal cycler (Bio-Rad) with initial denaturation at 94 °C for 3 min, followed by 30 cycles of denaturation at 94 °C for 45 s, annealing at 55 °C for 1 min, extension at 72 °C for 3 min with a final extension of 72 °C for 10 min. The PCR products were analyzed by electrophoresis in 1% low melting point agarose gel with ethidium bromide. The amplicons were purified using a GeneJET Gel extraction kit (ThermoFisher Scientific, USA) and were cloned directionally into the pET15b vector (Novagen, Madison, USA) at the *Nde1* and *BamHI* restriction sites. The recombinant clones selected on Luria broth (LB) agar plates containing ampicillin (50 µg/mL) were verified by colony PCR, restriction enzyme analysis and sequencing by universal T7 primers.

### 2.2. Expression and optimization of VEGF121 protein 

The pETVEGF recombinant plasmid was transformed into *E. coli* BL21 pLysS cells, and the individual colonies were grown in LB medium at 37 °C until the culture reached the midlog phase (OD600 nm of 0.4–0.5). The expression was induced at 37 °C by using 1 mM isopropyl β-D-1-thiogalactopyranoside (IPTG, Fermentas, USA) for 5 h. The temperature, IPTG concentration, solubility, and the hour interval for the expression of foreign proteins in *E. coli* BL21 pLysS were carried out as per the procedures described by Jia et al. (Jia et al., 2007). Induced samples were collected and analyzed by sodium dodecyl sulfate-polyacrylamide gel electrophoresis (SDS-PAGE) as per standard procedures.

### 2.3. VEGF121 purification and refolding

An *E. coli* pellet expressing VEGF121 was suspended in 5 mL of solubilisation buffer (20 mM Tris HCl pH8.0, 500 mM NaCl, 8 M urea, 5mM βME, 0.5% Triton X-100, 1 mM EDTA, 100 mM phenylmethylsulfonyl fluoride (PMSF), and 0.5 mg/mL lysozyme) and sonicated for cell disruption (Sonics Vibra-Cell VC 750 Sonicator) for 10 min at a burst speed of 40% amplitude with a repetitive on/off cycle for 9 s. The sonicated samples were further centrifuged at 17,696 × *g* for 15 min. The insoluble recombinant VEGF was purified using nickel affinity chromatography with the binding of the N-terminal hexa-histidine tag. The protein was bound to a column with repeated passing and the column was washed with solubilization buffer with 20 mM imidazole. The recombinant VEGF121 (rVEGF121) was eluted with 300 mM imidazole in the elution buffer (solubilization buffer with imidazole). The eluted fractions of protein were pooled and dialyzed in the dialysis buffer (25 mM Tris HCl pH 8, 1 mm EDTA, 1 mM DTT, 5% glycerol) containing between 8 M to 0 M urea, which was routinely changed until precipitation occured and further dialysis was carried out without urea to refold the protein in a soluble form for its suitability in bioassays. The purified proteins were assessed by SDS-PAGE and confirmed by Western blot and the concentration was estimated using the Bicinchoninic acid (BCA) method (Boster Biological Technology, CA, USA). 

### 2.4. SDS-PAGE and western blot analysis

The production of rVEGF121 along with harvested and eluted fractions were mixed separately with SDS sample buffer and resolved by polyacrylamide gel electrophoresis (PAGE) (12% resolving). The separated proteins done by SDS-PAGE were transferred onto a Polyvinylidene difluoride (PVDF) membrane (Bio-Rad) by Trans-Blot Turbo Transfer System (Bio-Rad) at 25 volts for 7 min. The membrane was blocked with 5% skim milk powder in phosphate-buffered saline (PBS) overnight at 4 °C. The rVEGF121 on the blot was detected by incubation with Bevacizumab (Avastin, commercial anti-VEGF, at a dilution of 1:10000) as primary antibody for 1 h at room temperature. Then the blot was incubated with an antihuman IgG HRP-conjugated (at a dilution of 1:10,000) as a secondary antibody for 45 min. The peroxidase activity was detected using the ChemiDoc Touch Imaging System (Bio-Rad).

### 2.5. Preparation of VEGF-containing microparticles

Microparticles containing VEGF was prepared by double emulsion solvent evaporation method (w/o/w) as described by in the literature with slight modifications (Anugraha et al., 2015; Rui et al., 2012). Briefly, 5 µg of rVEGF121 was dissolved separately in 0.25 mL of 10% polyvinyl alcohol (w/v) (PVA) (M.W. 30 to 70 kDa; 87%–90% hydrolyzed) (Sigma-Aldrich, Bangalore, India) and 100 mg of polylactide (M.W. 75–120 kDa (Sigma-Aldrich, Bangalore, India) in 2 mL of dichloromethane (50 mg/mL) was separately prepared. Both mixtures pooled together and sonicated for 2 min at a burst speed of 40% amplitude with a repetitive on/off cycle for 9 s. The emulsion was slowly added into 75 mL of 5% PVA under vigorous agitation in a magnetic stirrer. The microparticles formed through overnight solvent evaporation were centrifuged (Thermo scientific SORVALL LYNX 4000 centrifuge) at 10,000 × *g*/20 min/4 °C. The centrifugation was repeated twice with sterile distilled water to remove any surfactants. The pellet was lyophilized (Bench TopPro with Omnitronics Sp Scientific) overnight to form a free-flowing powder. The same way 200 µL of PBS was encapsulated with PLA to be used as a control. 

### 2.6. Characterization of VEGF microparticles

#### 2.6.1. Estimation of encapsulation efficiency

The polymer was solubilized by accurately weighing 10 mg of the microparticles by dissolving it in acetonitrile. The mixture was centrifuged at 10,000 × *g*/10 min at room temperature (RT), the supernatant was discarded, and the pellet was dried at 37 °C for an hour. Following this, 100 µL of PBS was added and centrifuged at 10,000 × *g*/10 min/RT. The supernatant was collected and the protein content was estimated by BCA as per manufacturer’s protocol (Boster Biological Technology, CA, USA). The absorbance was read at 562 nm. The percentage of encapsulation efficiency was calculated by the formula (µg of VEGF/mg of PLA particles * 100). 

#### 2.6.2. Scanning electron microscopy (SEM)

The particles were prepared by double emulsion, and the solvent evaporation method was subjected to scanning electron microscopy (SEM) for analysis of size and surface morphology (Carl ZEISS-EVO 18, Germany) using tungsten filament at an operating voltage of 10 kV. Approximately 10 µg of the sample was coated with gold atoms using a sputter coater (SC7620 ‘mini’ sputter coater and glow discharge system, to enhance scattering of electrons) with a vacuum pressure maintained at 1.32 × 10–5 mbar. The sample was loaded into the aluminum holder stub using tweezers and double-sided carbon tape. 

### 2.7. Release kinetics 

In vitro release of VEGF from PLA was determined in PBS at 37 °C. Briefly, 40 mg of the lyophilized powder was redispersed in 1-mL PBS in a 1.5 mL microcentrifuge tube, vortexed and centrifuged at 5422 × *g*/5 min, and the supernatant was collected (0 h). Fresh 1 mL of PBS was added, vortexed and placed in a 37 °C shaking incubator at 80 rpm in a slanting position. The supernatant was collected daily until day 30. After each collection, the supernatant was replaced with fresh 1-mL PBS and vortexed before being placed in 37 °C shaking incubator. After collection, the supernatant was always divided into 2 parts: one for analyzing release efficiency and the other for bioactivity assay. The same way PBS encapsulated with PLA was also treated and collected in a 24-h fraction for further experimental comparisons. All the supernatants were stored at –80 °C until use. 

### 2.8. SDS-PAGE analysis

The stability of the encapsulated microparticles was analyzed in SDS-PAGE. A known amount of protein VEGF121 encapsulated with PLA microparticles was incubated with 0.1% of SDS-PBS (0.01 M; pH 7.4) RT for 3 h by gentle shaking and then centrifuged at 20,584 × *g* for 25 min at 4 °C (Saini et al., 2011). The respective supernatants were mixed with Laemmli buffer, heated at 95 °C, and analyzed in SDS-PAGE. 

### 2.9. Microparticles release efficiency 

The amount of VEGF encapsulated at each time point was quantified by Enzyme Linked Immunosorbent Assay (ELISA). Supernatant from all time points was prethawed at 4 °C before use and measured by ELISA, according to the manufacturer’s instructions (Human VEGF PicoKine ELISA Kit, Boster Bio, CA, USA). Exactly 0.1 mL of the supernatant from each time point (1 to 30 days) was added to the precoated 96-well plate. The plate was incubated at 37 °C for 90 min. The plate content was then discarded, and 0.1 mL of the biotinylated antihuman VEGF antibody was added to all of the wells and incubated at 37 °C for 60 min. The plates were washed thrice with wash buffer. After washing the microtitre plate, 0.1 mL of Avidin-Biotin-Peroxidase Complex was added into each well and incubated at 37 °C for 30 min. Plates were washed 5 times with washing buffer followed by addition of 90 µL of the substrate into each well and incubated at 37 °C in the dark for 20 min. Once the color started developing, 0.1 mL of stop solution was added and the OD was determined at 450 nm within 30 min in a microplate reader (Spectramax M5e, Molecular Devices).

### 2.10. Bioactivity of rVEGF121 and VEGF-loaded PLA microparticles

#### 2.10.1. Proliferation and migration assay

Human umbilical vein endothelial cells (HUVEC) were purchased from HiMedia Laboratories (Mumbai, India). All experiments were performed using the cells below passage 8. Cells were grown in HiEndoXLTM Endothelial Cell Expansion Medium, Reduced Serum (HiMedia, Mumbai, India). 5000 cells/well were cultivated in 96 well plates (Corning, NY, USA), in the specified medium and were incubated in 5% CO2 incubator for the cells to adhere to the wells. The medium was later replaced with fresh serum and growth factor reduced medium containing rVEGF121 (200 and 500 ng), 100 µL of VEGF-loaded PLA microparticles (days 1, 20, and 30), and 100 µL of PBS-loaded PLA microparticles. Following this, its effects on proliferation were determined, and 10 ng/mL of commercially available recombinant VEGF121 (Sino Biological, MD, USA) was used as a positive control. MTT assay was performed as per the manufacturer’s protocol (HiMedia, Mumbai, India), purple formazan was used as an indicator, and the plate was spectrophotometrically measured at 570 nm with an ELISA plate reader (Spectramax M5e, Molecular Devices). 

For migration assay, 5000 cells/well (Corning, NY, USA) were seeded in 24-well plates and grown until a monolayer was formed. The monolayer was scratched with a sterile tip, the cellular debris was washed with PBS, and the cells were thereafter incubated with and without rVEGF121 (200 and 500 ng) for 12 h. Next, 100 µL of VEGF-loaded PLA microparticles (day 1, 20, and 30) and PBS-loaded PLA microparticles in serum-free RPMI medium were incubated for 24 h. Cell images were captured at a magnification of 4× in a camera connected with an inverted microscope (Leica DMi1). Image J was used to determine the wound healing percentage. 

#### 2.10.2. Endothelial tube formation assay

Approximately 96 well plates were coated with 50 µL/well of precooled Matrigel (BD Biosciences) and incubated in a humidified incubator (37 °C, 5% CO2) for 30 min for the Matrigel to solidify. Then, 1 × 104 cells/well were seeded with and without rVEGF121 (200 and 500 ng), 100µL of VEGF-loaded PLA microparticles (day 1, 20, and 30), and PBS-loaded PLA microparticles (negative control) and incubated for 4 h. After the incubation, CalceinAM (Trevigen), a fluorescent monitoring tube formation and a cell-permeable dye were added at a final concentration of 2 µg/mL and incubated for 30 min at 37 °C and provided with 5% CO2 in a light-free environment. The cell conditions and tube formation were monitored under a Nikon Laser Scanning Confocal microscope model C2 at 4× magnification, and the image was captured in the camera provided with the microscope. The number of tubes formed were analyzed using WimCam online software (Wimasis, Onimagin Technologies SCA, Spain). 

#### 2.10.3. Chicken chorioallantoic membrane assay 

Fresh embryonated eggs were purchased from the College of Poultry Production and Management (CPPM) in Hosur, India. The eggs were sterilized with 70% ethanol and incubated at 37 °C at 60%–70% humidity. After 4 days, the window of the embryonated chicken eggs was opened by removing the shell membrane. Filter paper discs soaked in PBS-PLA (negative control), rVEGF121 with 200 ng/mL and 500 ng/mL or VEGF-loaded PLA particles (day 1, 20, and 30) with 10–100 ng/mL were applied to the CAM individually, and the windows were sealed with parafilm and incubated for 24 to 96 h. The live blood vessel formation was recorded and photographed with a digital camera at 10× magnification after 48 h. The response and the total vessels network length of the blood vessels were quantified with WimCAM online software (Wimasis, Onimagin Technologies SCA, Spain)

### 2.11. Statistical analysis

The numerical data was represented as mean ± SD. Statistical significance was analyzed using a Bonferroni post-test using Graph Pad Prism, data analysis, and graphical software. Probability (P) value was defined as P < 0.05 and considered statistically significant, which is indicated with asterisks. 

## 3. Results

### 3.1. Amplification and cloning of isoform in pET 15b vector

**Figure 1:**
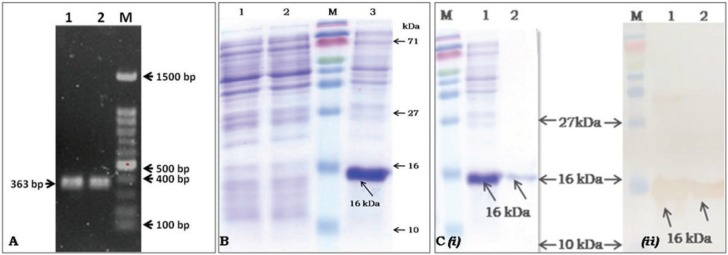
Cloning, expression, and western blotting analysis of VEGF 121: A. Agarose Gel Electrophoresis of the amplified VEGF 121 region from HESC: Lane 1 and 2: amplified VEGF 121 region; Lane 3: DNA ladder marker dye plus (TAKARA BIO INC.); B. SDS- PAGE analysis expressed rVEGF 121: Lane 1: pET 15b vector control; Lane 2: pETVEGF, uninduced protein control; Lane M: prestained protein ladder plus (Purogene, Genetix, New Delhi); Lane 3: pETVEGF, IPTG induced crude protein showing 16 kDa thick band, which is absent in first two lanes; C. SDS-PAGE and western blot analysis for the purified rVEGF 121 protein: (i) SDS-PAGE (ii); Western blot analysis; Lane M: prestained protein ladder plus (Purogene, Genetix, New Delhi); Lane 1: crude pETVEGF; Lane 4: purified pETVEGF.

The VEGF121 gene sequence was amplified from HESC with the gene-specific primers (Figure 1A). VEGF121 was excised, purified and ligated into the pET15b vector at the *NdeI *and *BamHI *sites. The *E. coli* DH5α cells were transformed with the ligated mixture and the transformants were screened by ampicillin selection. Presence of the gene-363 bp was confirmed by PCR and restriction digestion with *NdeI *and *BamHI* enzymes (data not shown). The ORF of the insert with N-terminal His tag was confirmed by sequencing with T7 universal primers.

### 3.2. Heterologous protein expression and optimization

The positive clones were transformed into BL21 (DE3) pLysS and analyzed in SDS-PAGE (Figure 1B). A single intense thick band was observed at 16 kDa (Figure 1B, lane 3) corresponding to rVEGF121 whereas no proteins were seen in the vector or uninduced controls (Figure 1B, lanes 1 and 2). The induction conditions were optimized by postinduction temperature (25, 30, 35, and 37 °C). Various inducer concentrations of IPTG (0, 0.5, 1, 1.5, and 2 mM) and different period of induction times (0, 1, 2, 3, 4, 5, 6, 7, 8, 9, and 10 h) were compared with one another by the simple analysis of expressed protein in SDS-PAGE (data not shown). However, rVEGF121 was better expressed at 37 °C in *E. coli* BL21 (DE3) pLysS cells; subsequent expression was seen up to 10 h of induction. The optimized duration of 5 h induction with 1 mM IPTG concentration at 37 °C incubation was chosen for further studies. 

### 3.3. Purification, refolding, and characterization of rVEGF121 protein

Rapid purification of the inclusion body fractions was performed under denaturing conditions at RT using His-tagged Ni-NTA resin (Qiagen, Germany). The unbound proteins were washed and the His-tagged VEGF121 protein was recovered by elution with 300 mM imidazole. Eluted fractions containing the expected single rVEGF121 protein were pooled and dialyzed for renaturation at 4 °C in different urea concentrations from 8 M, 6 M, 4 M, 2 M, 1 M, and 0.5 M. The precipitation was observed in 0.5 M urea and was further dialyzed without urea to bring the refolded protein into a solubilized one. The protein was confirmed by Western blot analysis (Figure 1C; (i) and (ii)) using Bevacizumab as a primary detection antibody. 

### 3.4. Characterization of rVEGF121 loaded PLA microparticle

**Figure 2:**
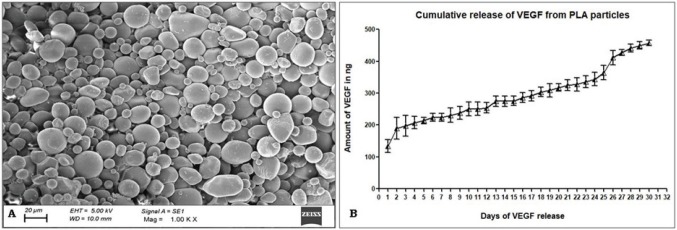
Characterization of rVEGF121-loaded PLA microparticle: a) Scanning Electron Microscopy (SEM) image of rVEGF121 encapsulated by PLA microparticles (size, and morphology of the particles were analysed using image J); b) In vitro release of rVEGF121 entrapped from PLA microparticles. In vitro release of rVEGF121 from VEGF-PLA microparticles released at 24 h intervals were determined.

SEM analysis of PLA encapsulated rVEGF121 showed the formation of a stable layer and spherical shape with smooth surfaces (Figure 2A). This indicates that VEGF121 did not form aggregation, and the size of the VEGF121 entrapped PLA particles were from 11.2 ± 1.7 to 23.9 ± 3.08 µm. The percentage encapsulation efficiency by BCA method of the microparticle was found to be 75%. 

### 3.5. In vitro drug release assay 

The quantification of rVEGF121 was done by incubating PLA microparticles in PBS at 37 °C for 30 days. ELISA was used to estimate VEGF121 (Figure 2B) present in the supernatant collected. Initially, there was a burst release of microparticles, which could be due to the deposition of protein on the surface of the microparticles. There were sustained release rates observed from day 1 to day 30. The cumulative release percentage at the end of the assay was calculated as 30.5 ± 0.58%. 

### 3.6. Stability of the protein 

**Figure 3:**
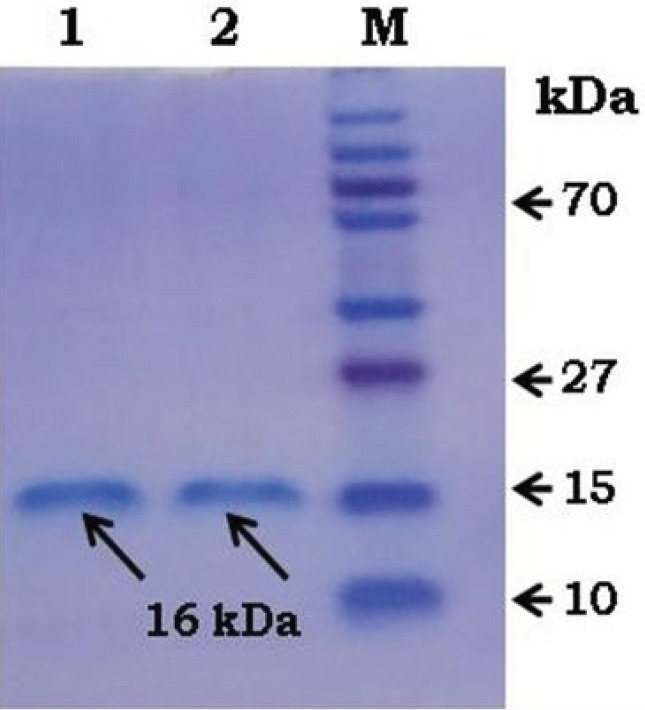
SDS-PAGE profile of PLA encapsulated microparticles: Lane 1: purified rVEGF121 (the arrow indicating 16 kDa); Lane 2: rVEGF121 released in vitro from PLA encapsulated microparticles (the arrow indicating 16 kDa); M: prestained protein ladder plus (Page Ruler, Thermo Scientific).

The purified rVEGF121 protein and the in vitro released protein from PLA microparticles were run in SDS-PAGE. The resolved bands of the released protein from PLA particles revealed that microparticle preparation conditions did not cause any degradation of the protein (Figure 3), and the protein was found to be stable. 

### 3.7. In vitro proliferation assay

The proliferation of rVEGF121 and VEGF-PLA particles were assessed in HUVEC cells. To evaluate the functionality of the recombinant VEGF121, the cells were treated with 200 ng and 500 ng of rVEGF121 (Figure 4B) and positive control. Recombinant VEGF121 and VEGF-PLA particles significantly enhanced the HUVEC cells proliferation (P < 0.0001). Cells treated with 500 ng and rVEGF121 showed a significant proliferation of 43.51% compared to the cells treated with 200 ng of rVEGF121 (35.04%). Meanwhile, cells treated with VEGF121 microparticles collected at time points (days 1, 20, and 30) showed significant proliferation (P < 0.0001) at 41.36%, 17.76%, and 12.40%, respectively, when compared with PBS-loaded PLA microparticles (3.10%) (Figure 4B). 

**Figure 4A:**
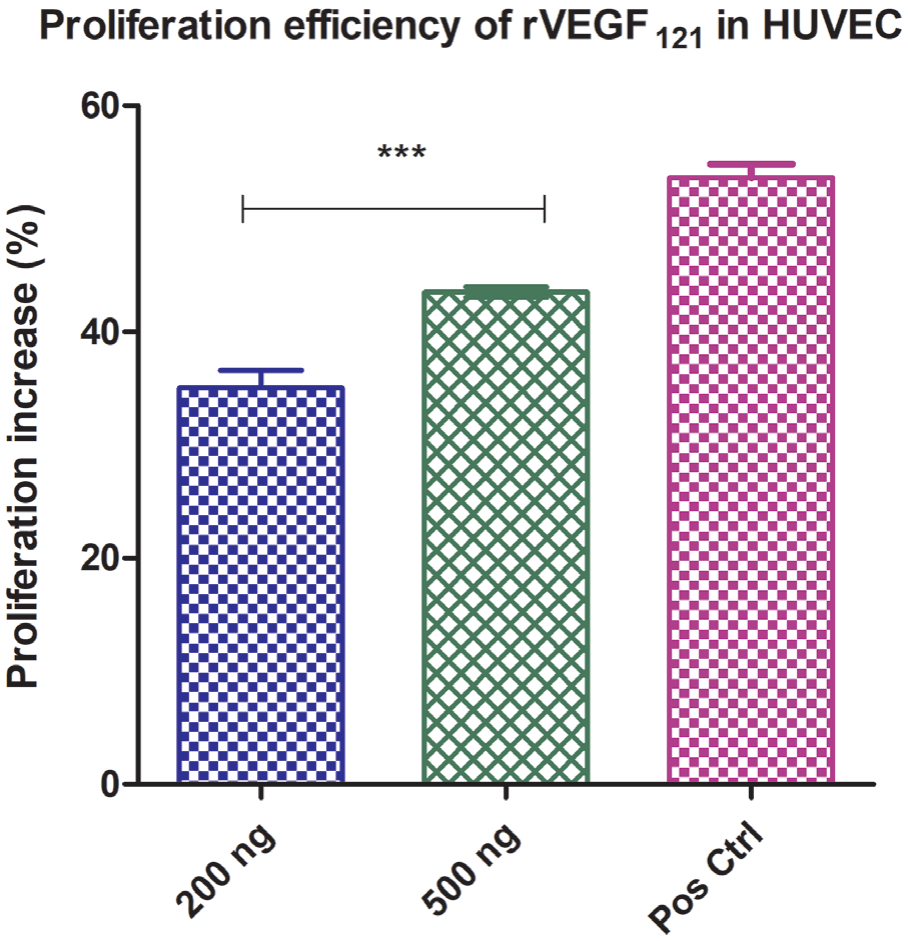
In vitro proliferation assay in HUVEC cells: MTT proliferation assay of rVEGF121. HUVEC cells were incubated with various concentrations of rVEGF121 for 24 h and compared to the effects with control (serum free RPMI). Absorbance read at 570 nm; the data represented as mean ± S.D. from 3 replicates. The Y-axis shows the percentage of proliferation increase, normalized to control cells. Asterisks (***) indicate significant differences of P < 0.0001 for 500 ng vs. 200 ng.

**Figure 4B:**
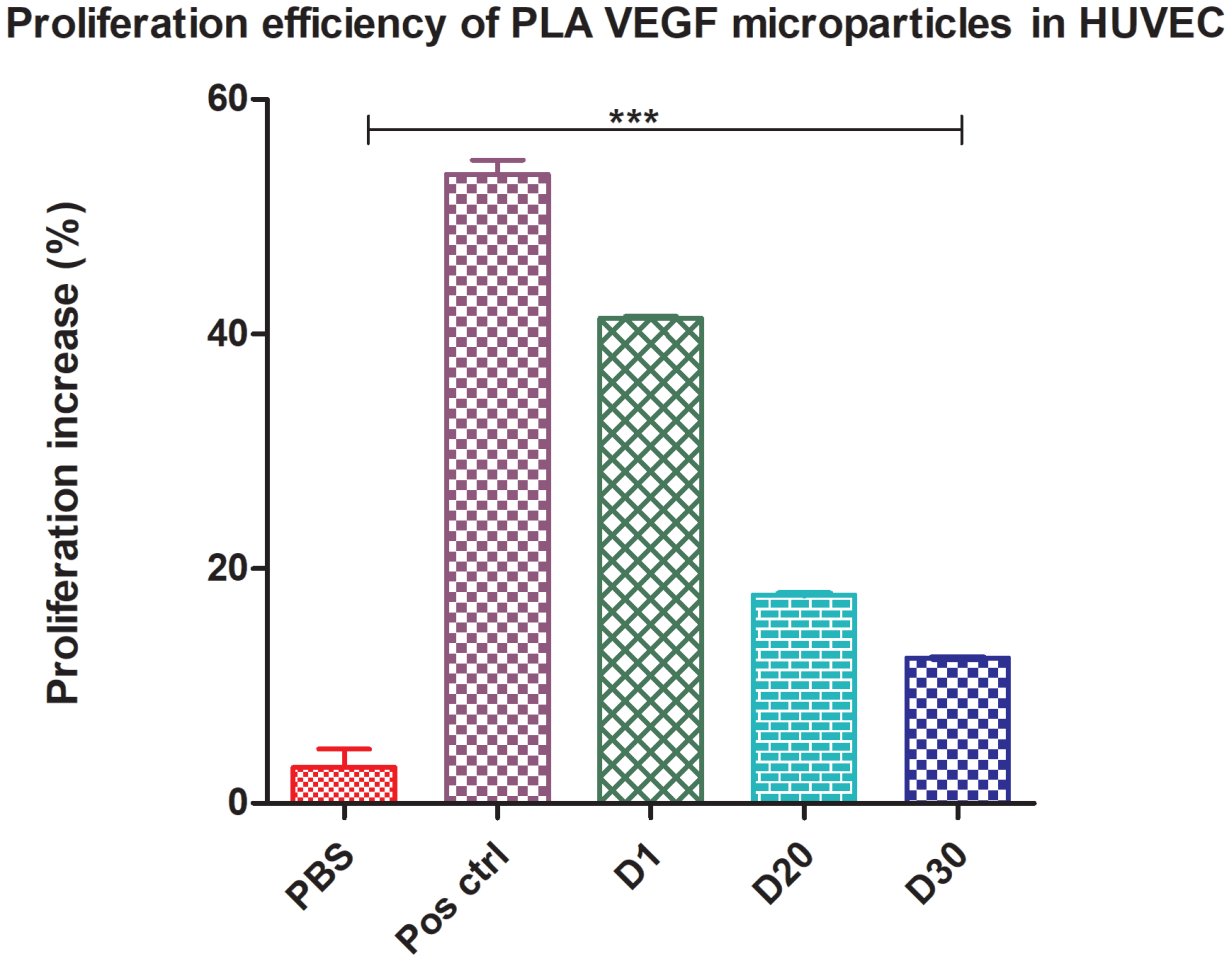
In vitro proliferation assay in HUVEC cells. MTT proliferation response of PLA encapsulated rVEGF121. HUVEC cells incubated with various concentrations for 24 h and compared with the negative control and positive control (500 ng of rVEGF121). Absorbance read at 570 nm; the data represented as mean ± S.D. from 3 replicates. The Y-axis shows the percentage proliferation increase, normalized to control cells. Asterisks (***) indicate significant differences of P < 0.0001 for PBS (negative control) vs. day 1, day 20, and day 30.

### 3.8. In vitro wound healing migration assay

Cells treated with recombinant VEGF121 and the rVEGF121-loaded microparticles were employed in wound healing assays. Recombinant VEGF121 facilitated significant healing of the wound at 12 h (Figure 5A) compared to the untreated control (100% in 500 ng of rVEGF121 and 92% in 200 ng rVEGF121) (Figure 5B). Even after 24 h, the untreated cells were not able to heal the wounds completely. Recombinant VEGF121 loaded microparticles were also able to heal the wounds but at a slower rate. Day 1 had 95% (which is equivalent to the cells treated with 500 ng rVEGF121) while day 20 and 30 had 83% and 82% migratory potential at 24 h. In contrast, the PBS-loaded PLA microparticle showed a percentage of 75.82%, which is similar or equal to the untreated control at 73.94%. Slow healing was observed continuously until 96 h. (Figure 5C). This migration assay confirms that rVEGF121 and the rVEGF121-loaded microparticles are biologically active. However, rVEGF121 heals the wound faster and VEGF121-loaded microparticles are slow and stable, confirming the efficiency for prolonged healing.

**Figure 5A:**
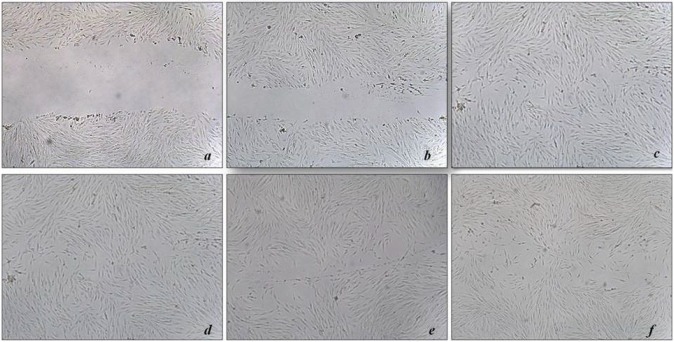
Effect of rVEGF121 and PLA–VEGF microparticles on HUVEC cells. Cell migration experiments were determined in wound healing scratch experiments. Images of the gap area induced at 12 h: a) Untreated cells at 12 h; b) 200 ng of rVEGF121 at 12 h; c) 500 ng of rVEGF121 at 12 h; d) PLA–VEGF microparticles on day 1 at 24 h; e) PLA–VEGF microparticles on day 20 at 24 h; f) PLA–VEGF microparticles on day 30 at 24 h.

**Figure 5B:**
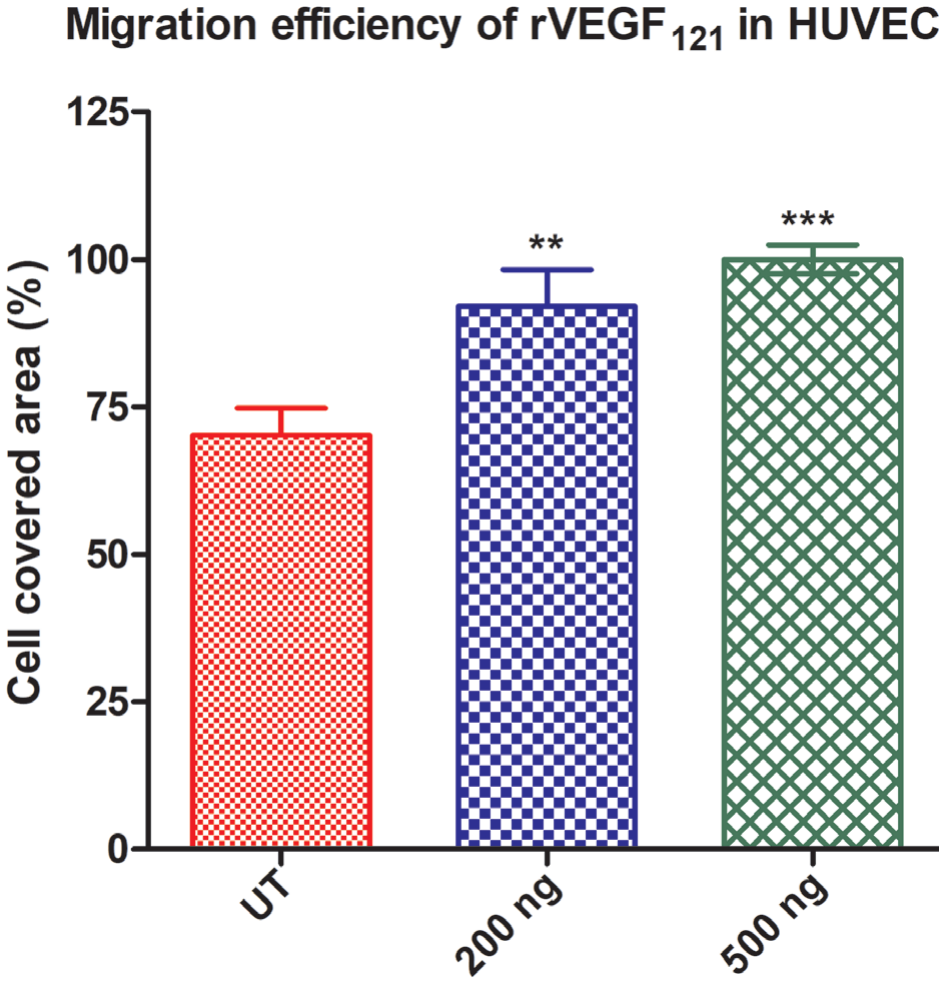
Effect of rVEGF121 microparticles on HUVEC cells. The histogram shows the percentage of cell-covered area for different concentration of rVEGF121. Percentage effects were compared with the untreated control (UT). Data represented (mean ± S.D., n = 3) at 12h. Statistical significance shown is 200 ng and 500 ng vs. untreated group.

**Figure 5C:**
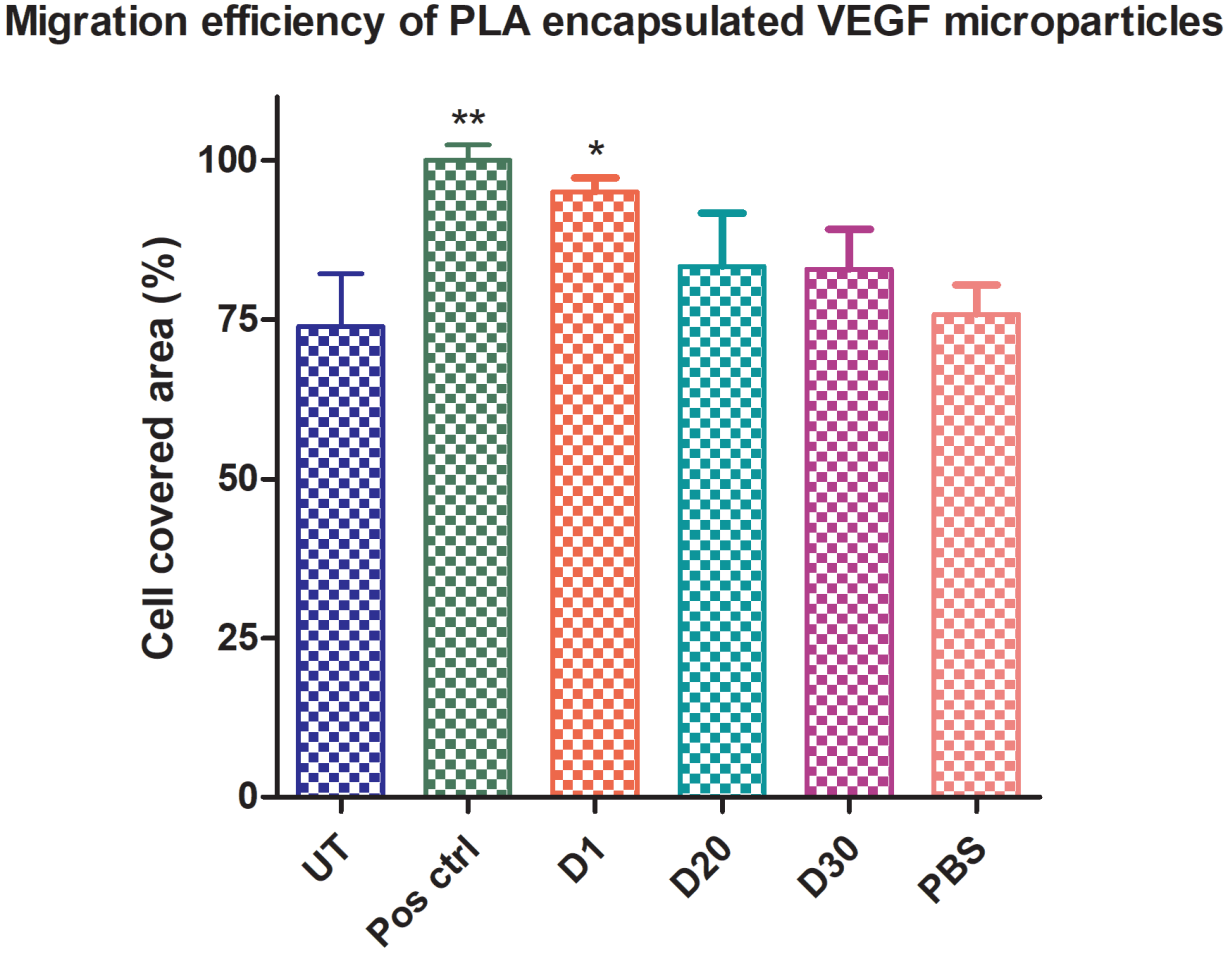
Effect of rVEGF121 loaded microparticles on HUVEC cells. Histogram shows the percentage of cell-covered area at 24 h treated with rVEGF121-loaded microparticles collected on different days. Data represented as mean ± S.D., n = 3. Statistical significance compared with PBS-loaded PLA microparticle (negative control).

### 3.9. Endothelial tube formation assay 

HUVEC cells treated with rVEGF121 and rVEGF121-loaded microparticles have the ability to form a tube-like structure. The number of endothelial tubes formed was measured in cells treated with rVEGF121 (200 and 500 ng) and rVEGF121-loaded microparticles (day 1, 20, and 30), which were compared with a PBS-loaded PLA microparticle (negative control). The number of tubes formed was significantly greater than the negative control (Figure 6A). While the highest number of tubes observed were in 500 ng (i.e. 113 ± 1.41, followed by 108 ± 2.82 for 200 ng concentration), day 1 samples of rVEGF121-loaded microparticles formed 105 ± 1.41, followed by 51 ± 2.82 and 49 ± 1.41 for days 20 and 30, respectively (Figure 6B). Analysis was done using Wimasis Image Analysis software (Wimasis, 2016a). 

**Figure 6A:**
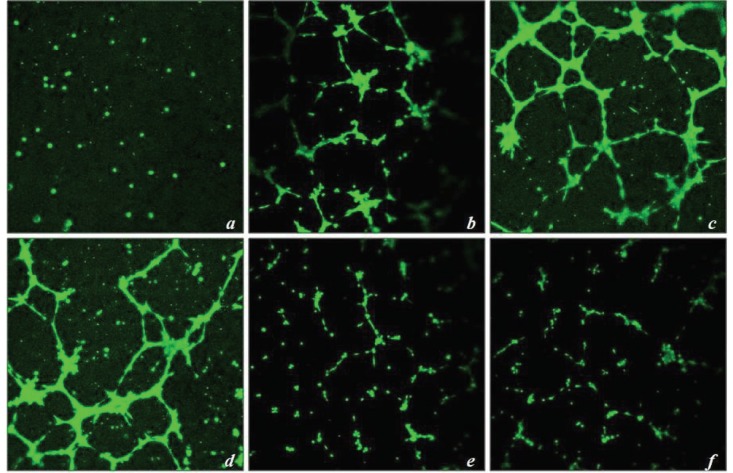
Effect of rVEGF121 and rVEGF121-loaded microparticles on tube formation. Images indicate the endothelial tube formation after 4 h of incubation in HUVEC cells: a) PBS-PLA (negative control); b) 200 ng of rVEGF121; c) 500 ng of rVEGF121; d) PLA–VEGF microparticles on day 1; e) PLA–VEGF microparticles on day 20; f) PLA–VEGF microparticles on day 30. Photographs were taken at a 20× magnification under a confocal microscope.

**Figure 6B:**
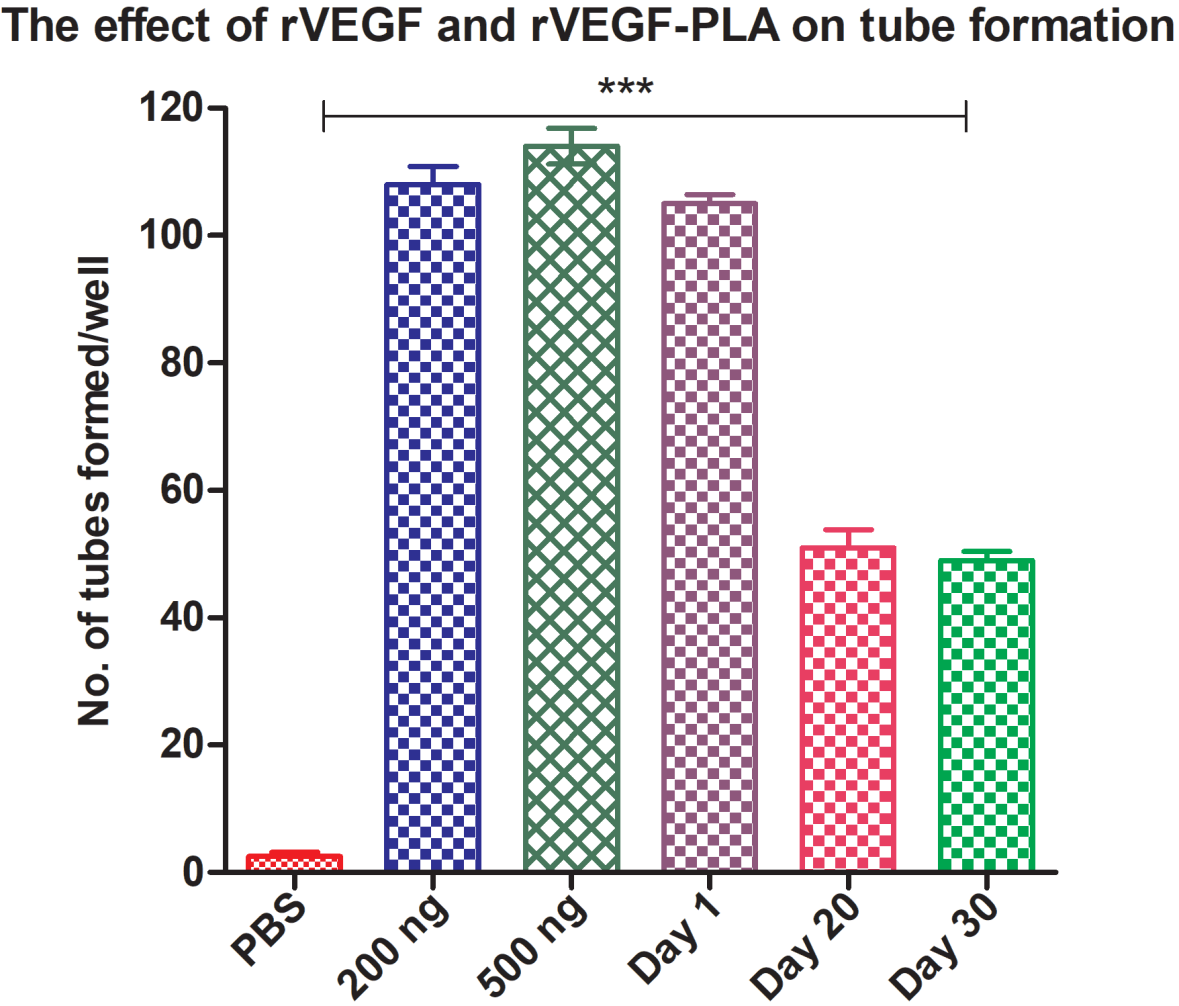
Effect of rVEGF121 and PLA–VEGF microparticles on tube formation. Graphs show the number of endothelial tubes formed by HUVEC cells exposed to PBS-PLA, different concentrations of rVEGF121 and PLA–VEGF microparticles collected at different days for 4 h in Matrigel-coated 96 well plates. Data presented as mean ± S.D with n = 3. Asterisks (***) denote statistically significant differences compared to PBS-loaded PLA microparticles (negative control).

### 3.10. Chicken chorioallantoic membrane assay 

Recombinant VEGF121, rVEGF121-loaded microparticles and PBS-loaded PLA microparticle (negative control) were applied on third-day eggs for the evaluation of blood vessels to check the proangiogenic action of VEGF. New capillary blood vessels formed in all the groups after 48 h (Figure 7A). In the control groups (PBS-PLA), blood vessel formation was normal whereas in eggs applied with 200 and 500 ng rVEGF121, there was a significant increase in blood vessels; rVEGF121-loaded microparticles also had a significant increase in the formation of blood vessel network. (Figure 7B). Analysis was done with Wimasis Image Analysis software (Wimasis, 2016b). 

**Figure 7A:**
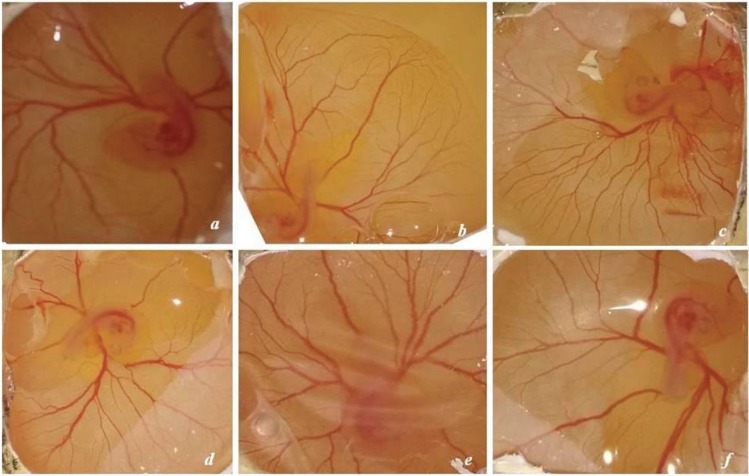
Effect of rVEGF121 and PLA–VEGF microparticles on ex vivo angiogenesis. Filter discs soaked in a) PBS-PLA (negative control); b) 200 ng of rVEGF121; c) 500 ng of rVEGF121; d) PLA–VEGF microparticles on day 1; e) PLA–VEGF microparticles on day 20; f) PLA–VEGF microparticles on day 30. CAMs were photographed at a magnification of 10× with digital camera after 48 h.

**Figure 7B:**
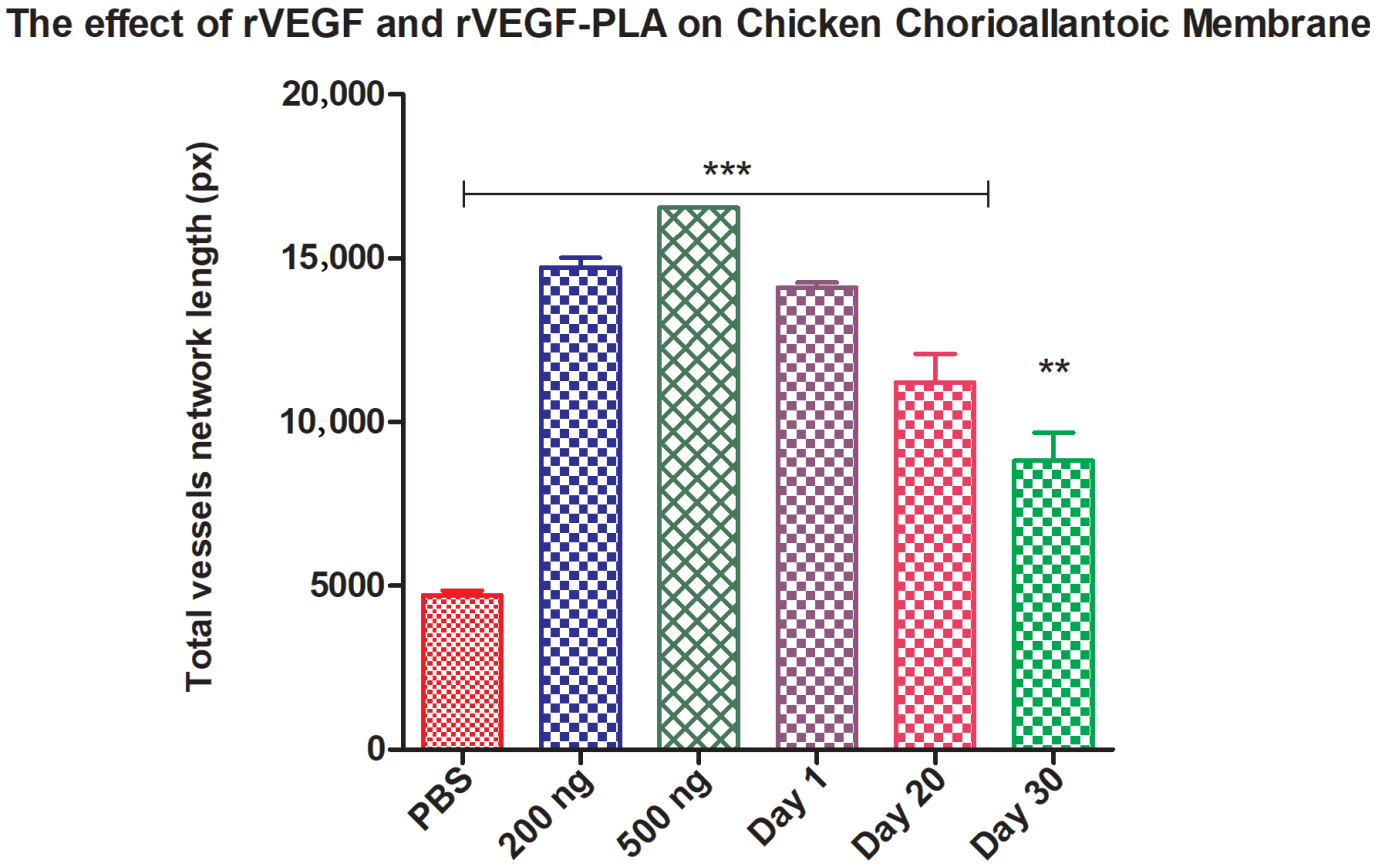
Effect of rVEGF121 and PLA–VEGF microparticles on ex vivo angiogenesis: graphical representation of the vessel length induced by different concentrations of rVEGF121 and PLA–VEGF microparticles collected on different days. Quantitative measurement of the total vessel network length was calculated; data is represented as mean ± S.D., n = 3. Statistical significance was compared with PBS (PBS-PLA) (negative control)

## 4. Discussion

Angiogenesis is the formation of new blood vessels with many molecules involved in the process. It occurs throughout life, and control of angiogenesis (antiangiogenesis) can be used in cancer therapies (Adair and Montani, 2010). Promotion (proangiogenesis) leads to regeneration therapies like endometrium, diabetic wound healing, and cardiovascular diseases. VEGF, VEGF-A with various isoforms expressed in many tissues, is one of the important growth factors involved in physiological angiogenesis, but an aberrant expression of VEGF leads to pathological angiogenesis (Shibuya, 2011). Hence, VEGF is often exploited in many clinical applications such as in cancer therapeutics or diagnostics. 

VEGF is very important in blood vessel formation in the kidneys, brain, liver, and lungs. VEGF and angiogenesis play an important role in endometrium regeneration (Sharkey et al., 2000; Smith, 2001). When studied for polymorphism, this gene has been linked to gynecologic and obstetric conditions like recurrent implantation failure (Jung et al., 2016). VEGF helps in endothelial cell migration; in a study by Beckert et al., lactate enhanced collagen synthesis influenced the production of endothelial cells and thereby increased cellular migration (Beckert et al., 2006). As discussed by Huang et al. VEGF121 was expressed in the endometrium as a 16 kDa protein in recombinant form (Kazemi-Lomedasht et al., 2014) which plays an important role in endometrium regeneration (Huang et al. 1998) used as a source of gene for cloning purpose.

We amplified the VEGF121 gene (363 bp), which was composed of about 121 amino acids and ligated with a bacterial expression vector (pET 15b) and sequenced after cloning in DH5α strain of *E. coli*. The *E.coli *system is exploited for high-level expression and functionally active, heterologous proteins (Kaur et al., 2018). There are several recombinant proteins produced in* *the* E.coli *expression system approved by the FDA for clinical use, including Humulin (RH Insulin), IntronA (interferon α2b), Glucagon, and Preos (parathyroid hormone) (Baeshen et al., 2015). Producing the recombinant proteins in *E.coli* has several advantages such as a shorter duration, faster growth, easy maintenance, easy scale-up, inexpensive growth media, and low cost (Rosano and Ceccarelli, 2014). The target of this study is to successfully express VEGF121 in *E.coli* and to check the bioactivity of rVEGF121, which could be used as a proangiogenic drug for sustained release using PLA polymer as a delivery system. 

The slow delivery system of this protein will be advantageous as VEGF121 has a shorter half-life of <90 min (Rui et al., 2012); therefore, the carrier should be effective in releasing VEGF in a controlled manner. PLA, approved by the FDA, is safe, biodegradable, and has a long-standing capacity (Simamora and Chern, 2006) and therefore used extensively in medical applications.

Drug delivery systems can effectively work at the target site and thus reduce the harmful effects caused due to multiple dosages of drugs. PLA particles encapsulated with VEGF have been successfully used as nanosustained release microspheres, which induced proliferation and differentiation of adipose-derived stem cells (He et al., 2018).** **VEGF165 during encapsulation with PLA microparticles stimulated vasculogenesis and angiogenesis in an acute myocardial ischemia-reperfusion rat model (Formiga et al. 2010). PLA encapsulation was carried out with the double emulsion solvent evaporation method (w/o/w) with the modifications from Rui at al. (Rui et al., 2012; Anugraha et al., 2015). The size of the PLA particles ranged from 11.2 to 23.9 µm. The amount of polymer, nature of the solvent, preparation methods, and stirring rate influences the size of the particle. As reported by Howie et al. and Anugraha et al., larger size particles have less effect and smaller size particles have chances of more positivity (Howie et al., 1993; Anugraha et al., 2015). Microspheres ranging from 10 to 200 µm diameters achieved optimal release as reported by Han et al (Han et al., 2016). The double emulsion method requires high loading of the samples, which may also result in protein degradation during the process of sonication, centrifugation, and also interactions between the protein and the polymer. This might have influenced the interaction efficiency and is the possible reason for having 75% encapsulation efficiency. The evaluation of the microparticles from the resolved bands in the SDS-PAGE proved that the protein is stable. Our results show a burst release of VEGF during the preliminary days, and this is because the encapsulated protein is distributed on the surface and they were released instantly when engrossed in PBS. ELISA and in vitro release kinetics provide an overall idea of the study.

The bioactivity of rVEGF121 and the VEGF-PLA microparticles were studied in HUVEC cells derived from the endothelium of veins from the umbilical cord. They are extensively used as a model in laboratories to characterize the function of endothelial cells (Park et al., 2006). Wound healing assays with different concentrations of rVEGF and VEGF-PLA microparticles suggest that VEGF stimulates angiogenesis and plays an important role in wound healing and repair mechanisms. This also correlates with the reported study by (Taktak-Ben Amar et al., 2017)**. **The wound-healing effect of VEGF-PLA microparticles were slower when compared with rVEGF121 and the control, but the day 1 released microparticles did not have any significant difference when compared with the 500 ng of rVEGF. However, all the particles were able to repair the wounds, suggesting that the microparticles are stable and bioactive whereas PBS-loaded PLA microparticles showed similar results to that of the untreated control. The obtained results also correlate with earlier findings (Bao et al., 2009; Taktak-Ben Amar et al., 2017). HUVEC proliferation studies also suggest that rVEGF121 and VEGF-PLA microparticles are bioactive and have the ability to promote endothelial cells in vitro. Wound healing is a multistep process that is controlled by the involvement of different cells (Tonks et al., 2003). While investigating the proliferation and migration of rVEGF121 and rVEGF-encapsulated microparticles in HUVEC cells, we found that rVEGF121 and rVEGF-encapsulated microparticles accelerate wound closure more quickly than the controls, indicating the stimulatory effect of the recombinant protein and the encapsulated particles. Biological activity was also characterized by endothelial tube formation assay; all the samples were able to form tubes when compared with the control. Tube formation assay has numerous advantages as it is inexpensive, not time-consuming, and quantifiable. This result correlates with various reports already available for VEGF and also has the reproducibility for VEGF and its loaded particles proving to be bioactive.  In vitro* *tube formation assay possesses strong activities stimulating angiogenesis in vitro*.* Recombinant VEGF121 and VEGF-PLA microparticles also help in endothelial cell migration and promote angiogenesis by forming new capillary blood vessels in the CAM model. The total vessel length significantly increased in the eggs when rVEGF121 and VEGF-PLA microparticles were used. CAM assay provides a scientific understanding and the capacity of the PLA microparticles on angiogenesis, cell proliferation, and migration. 

To conclude, the study described here is a simple approach for producing a successful, functionally bioactive rVEGF121 in a bacterial expression system and using it effectively for therapeutic applications through a proper sustained delivery mode. The results presented here are significant when compared with the negative control (untreated or PBS-PLA) Thus, the combined effects of PLA and rVEGF121 accelerate wound healing in vitro,* *which could be used for wound closure applications and as a proangiogenic drug on further in vivo* *confirmation. 

## Acknowledgments

The authors would like to thank Innov4Sight Health and Biomedical Systems for their collaboration and support during the study.
